# Overestimation of piperacillin/tazobactam resistance in *Escherichia coli* by disc diffusion and gradient strip methods

**DOI:** 10.1093/jac/dkaf304

**Published:** 2025-08-18

**Authors:** Stefano Mancini, Corinne E Schoenenberger, Natalia Kolesnik-Goldmann, Vladimira Hinic, Adrian Egli, Oliver Nolte

**Affiliations:** Institute of Medical Microbiology, University Zurich, Zurich, Switzerland; Institute of Medical Microbiology, University Zurich, Zurich, Switzerland; Institute of Medical Microbiology, University Zurich, Zurich, Switzerland; Institute of Medical Microbiology, University Zurich, Zurich, Switzerland; Institute of Medical Microbiology, University Zurich, Zurich, Switzerland; Institute of Medical Microbiology, University Zurich, Zurich, Switzerland

Piperacillin/tazobactam (TZP) is a broad-spectrum β-lactam/β-lactamase-inhibitor combination that can be used as a carbapenem-sparing option for treating *Escherichia coli* infections, including those caused by ESBL-producing strains. However, the presence of β-lactamases such as OXA-1—and to a lesser extent, other enzymes like TEM—can reduce TZP susceptibility by increasing MICs. Several studies have shown that, in such cases, MICs are generally close to the clinical breakpoints (CBPs).^1,2^ Variability in antimicrobial susceptibility testing (AST) results has been reported with the reference broth microdilution (BMD) method, as well as with non-reference methods such as disc diffusion (DD) and gradient strip tests (GSTs).^3–5^ The use of different CBPs, as defined by EUCAST (≤8 mg/L for susceptible) and CLSI (≤16 mg/L for susceptible), adds further complexity and contributes to the challenges of accurate AST for TZP. We aimed to evaluate the robustness of non-automated AST methods for TZP, including DD and GSTs, using a collection of 98 genetically characterized *E. coli* clinical isolates collected between 1 February 2014 and 31 December 2014 at the Institute of Medical Microbiology, University of Zürich. Their genetic features were identified by WGS and published in a previous study.^6^ This collection comprised 26 strains without plasmid-borne ESBL or OXA-1 (none), 25 strains carrying a CTX-M-type ESBL, 16 with OXA-1 and 31 strains harbouring both a CTX-M-type ESBL and OXA-1 (Table S1, available as Supplementary data at *JAC* Online). MICs were determined using the standard BMD method according to the EUCAST guidelines^7^ and the GST (Liofilchem^®^-MTS^™^ piperacillin/tazobactam 0.016/4–256/4 mg/L). Growth inhibition zones were measured using discs containing 100 µg piperacillin and 10 µg tazobactam (Liofilchem, Italy), as recommended by CLSI, and discs containing 30 µg piperacillin and 6 µg tazobactam (Liofilchem, Italy), as recommended by EUCAST.^8,9^  *E. coli* ATCC25922 was used as an internal quality control (Figure S1).

The MIC distribution of the tested isolates as determined with the standard BMD method ranged from 0.5 mg/L to 128 mg/L, with a MIC_50_ of 4 mg/L and a MIC_90_ of 16 mg/L (Figure 1). Based on the EUCAST interpretation guidelines, no strain without OXA-1 was classified as resistant, while 50% of *E. coli* strains with the OXA-1 gene and 80% producing both OXA-1 and CTX-M were deemed susceptible. In total, 70.2% of strains with OXA-1 were susceptible, mostly exhibiting MICs close to the CBP.

**Figure 1.**
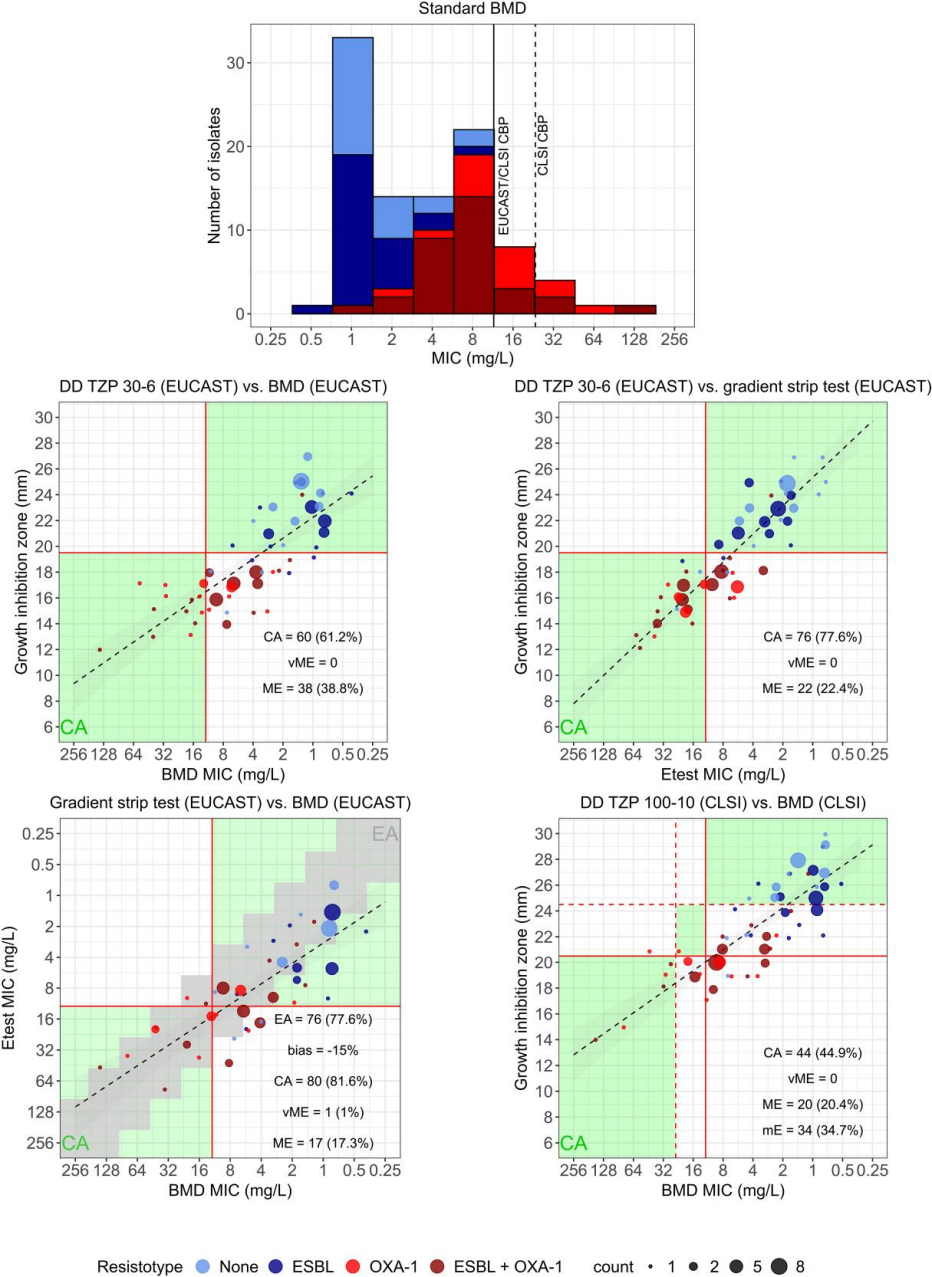
Piperacillin/tazobactam susceptibility testing. Top: Distribution of piperacillin/tazobactam MICs determined by the BMD method, stratified by β-lactamase type. Vertical lines indicate EUCAST and CLSI CBPs. Bottom: Correlation between disc diffusion and gradient strip testing compared with standard BMD. Vertical and horizontal lines represent EUCAST and CLSI CBPs. Green areas indicate categorical agreement; grey boxes indicate essential agreement.

Essential agreement (EA) and categorical agreement (CA) between the GST and the standard BMD method, both interpreted based on the EUCAST guidelines, were 77.6% and 81.6%, respectively, with a negative bias of −15%. Consistent with previous findings by Manuel *et al*.,^5^ most discrepancies were major errors (MEs, 17.3%), with only one very major error (vME) observed, indicating a tendency of the GST method to overestimate MICs. Similarly, EUCAST-based DD exhibited a CA of 61.2% with the EUCAST-based BMD method and resulted in 38 MEs (38.8%). Compared to the EUCAST-based GST, EUCAST-based DD showed a CA of 77.6%, with 22 MEs (22.4%), further corroborating the tendency of this method to overestimate resistance. Most MEs were observed among isolates harbouring OXA-1, reflecting the reduced susceptibility conferred by this enzyme to TZP. Finally, CLSI-based DD showed the lowest CA with the CLSI-based BMD method (44.9%) and resulted in 20 MEs and minor errors in 34.7% of cases, again indicating a general trend towards overcalling resistance with DD.

Using a large collection of *E. coli* clinical isolates producing β-lactamases known to affect TZP susceptibility, we confirmed previous findings that TZP resistance is likely driven by the (co-)production of OXA-1. Importantly, and in agreement with earlier studies, we also demonstrated that routine AST methods, such as DD and GST, lack robustness and tend to overestimate resistance, calling isolates ‘resistant’ when they are susceptible. It should be noted that our isolate collection was biased by an overrepresentation of OXA-1 producers, which likely exceeds the local prevalence. Nevertheless, our findings corroborate the notion that TZP resistance in *E. coli* may be systematically overcalled in clinical laboratories relying on DD and GSTs, regardless of whether EUCAST or CLSI guidelines are applied. In this context, Kahn *et al*. showed that a reduction in disc potency led to improved separation between susceptible and non-susceptible isolates.^4^

The use of TZP as a carbapenem-sparing option for invasive infections caused by ESBL-producing *E. coli* remains controversial, particularly following the MERINO study, which reported that carbapenems may be associated with lower 30-day mortality, especially in patients with terminal cancer. However, *post hoc* analysis has challenged this view, showing no significant difference in mortality when non-susceptible strains and those harbouring OXA-1 were excluded.^10^ This underscores the critical role of AST accuracy in guiding therapeutic decisions and influencing clinical outcomes.

While GSTs and DD methods tend to overestimate resistance to TZP—primarily resulting in MEs—most isolates categorized as susceptible by these methods are indeed likely to be truly susceptible. Consequently, the use of TZP could be considered appropriate in clinical practice when susceptibility is indicated by DD or GST.

## Supplementary Material

dkaf304_Supplementary_Data

## Supplementary data

Figure S1 and Table S1 are available as Supplementary data at *JAC* Online.
