# Supplementary material for: Overestimation of piperacillin/tazobactam resistance in Escherichia coli by disc diffusion and gradient strip methods
Source: J Antimicrob Chemother. 2025 Aug 18;80(12):3464–7. doi: 10.1093/jac/dkaf304 (PMC12670158; doi:10.1093/jac/dkaf304)
Supplement: dkaf304_Supplementary_Data [file dkaf304_supplementary_data.docx]

**Supplementary data**

**Table S1. Phenotypic and genetic features of the isolates used in this study.**

| **Study n.^1^** | **BMD** | **DD EUCAST** | **DD CLSI** | **Gradient**  **strip test** | **Plasmid-borne-beta-lactamase(s)** | **Beta-lactamase group** |
| --- | --- | --- | --- | --- | --- | --- |
| 2 | 1 | 21 | 25 | 3 | CTX-M-1 / TEM-1 | ESBL |
| 3 | 1 | 23 | 27 | 2 | SHV-1 / TEM-1 | None |
| 4 | 1 | 24 | 25 | 2 | CTX-M-1 / TEM-1 | ESBL |
| 7 | 4 | 19 | 22 | 12 | CTX-M-2 / CTX-M-8 / TEM-1 | ESBL |
| 8 | 1 | 22 | 25 | 3 | CTX-M-1 | ESBL |
| 9 | 1 | 22 | 24 | 3 | CTX-M-1 / TEM-1 | ESBL |
| 10 | 128 | 12 | 14 | 48 | CTX-M-15 / OXA-1 | ESBL + OXA-1 |
| 12 | 1 | 24 | 27 | 1.5 | CTX-M-15 / OXA-1 | ESBL + OXA-1 |
| 13 | 8 | 16 | 20 | 16 | CTX-M-1 / OXA-1 | ESBL + OXA-1 |
| 14 | 4 | 18 | 21 | 12 | CTX-M-1 / OXA-1 | ESBL + OXA-1 |
| 16 | 16 | 16 | 19 | 32 | CTX-M-15 / OXA-1 | ESBL + OXA-1 |
| 18 | 8 | 14 | 18 | 24 | CTX-M-15 / OXA-1 | ESBL + OXA-1 |
| 20 | 32 | 13 | 20 | 48 | CTX-M-1 / OXA-1 | ESBL + OXA-1 |
| 21 | 4 | 18 | 23 | 8 | CTX-M-1 / OXA-1 | ESBL + OXA-1 |
| 22 | 1 | 23 | 24 | 2 | CTX-M-1 / SHV-1 | ESBL |
| 25 | 2 | 18 | 22 | 8 | CTX-M-15 / TEM-1 | ESBL |
| 26 | 1 | 25 | 23 | 4 | CTX-M-15 / SHV/ TEM-1 | ESBL |
| 28 | 1 | 23 | 25 | 2 | CTX-M-15 / TEM-1 | ESBL |
| 29 | 1 | 23 | 26 | 2 | CTX-M-55 / TEM-1 | ESBL |
| 30 | 1 | 21 | 24 | 4 | CTX-M-9 / TEM-1 | ESBL |
| 32 | 1 | 22 | 26 | 3 | CTX-M-15 / TEM-1 | ESBL |
| 36 | 0.5 | 24 | 26 | 1.5 | CTX-M-14 / TEM-1 | ESBL |
| 37 | 1 | 19 | 22 | 6 | CTX-M-1 / TEM-1 | ESBL |
| 38 | 1 | 20 | 27 | 2 | CTX-M-15 / TEM-1 | ESBL |
| 39 | 2 | 20 | 24 | 6 | CTX-M-9 | ESBL |
| 40 | 2 | 18 | 21 | 4 | OXA-1 | OXA-1 |
| 42 | 1 | 23 | 27 | 2 | CTX-M-15 | ESBL |
| 43 | 1 | 22 | 25 | 2 | CTX-M-9 / TEM-1 | ESBL |
| 47 | 1 | 23 | 27 | 2 | CTX-M-9 / TEM-1 | ESBL |
| 51 | 4 | 17 | 20 | 12 | CTX-M-15 / OXA-1 | ESBL + OXA-1 |
| 57 | 2 | 21 | 25 | 3 | CTX-M-55 / TEM-1 | ESBL |
| 62 | 2 | 25 | 27 | 3 | TEM-1 | None |
| 63 | 2 | 19 | 24 | 6 | CTX-M-15 / OXA-1 | ESBL + OXA-1 |
| 66 | 4 | 15 | 19 | 16 | CTX-M-15 / OXA-1 / TEM-1 | ESBL + OXA-1 |
| 68 | 8 | 18 | 21 | 8 | CTX-M-15 / OXA-1 | ESBL + OXA-1 |
| 69 | 1 | 25 | 29 | 1.5 | CTX-M-27 | ESBL |
| 70 | 4 | 17 | 21 | 6 | CTX-M-15 / OXA-1 | ESBL + OXA-1 |
| 71 | 2 | 21 | 25 | 4 | CTX-M-15 / TEM-1 | ESBL |
| 74 | 8 | 17 | 20 | 12 | CTX-M-15 / OXA-1 | ESBL + OXA-1 |
| 75 | 8 | 16 | 20 | 12 | CTX-M-15 / OXA-1 | ESBL + OXA-1 |
| 76 | 4 | 17 | 20 | 12 | CTX-M-1 / OXA-1 | ESBL + OXA-1 |
| 82 | 16 | 15 | 19 | 24 | CTX-M-1 / OXA-1 / TEM-1 | ESBL + OXA-1 |
| 83 | 1 | 22 | 25 | 1.5 | CTX-M-15 / TEM-1 | ESBL |
| 86 | 8 | 18 | 21 | 8 | CTX-M-15 / OXA-1 | ESBL + OXA-1 |
| 89 | 8 | 14 | 18 | 32 | CTX-M-15 / OXA-1 / TEM-1 | ESBL + OXA-1 |
| 91 | 4 | 18 | 21 | 8 | CTX-M-15 / OXA-1 | ESBL + OXA-1 |
| 92 | 8 | 16 | 20 | 16 | CTX-M-15 / OXA-1 | ESBL + OXA-1 |
| 93 | 4 | 18 | 22 | 6 | CTX-M-15 / OXA-1 | ESBL + OXA-1 |
| 94 | 32 | 15 | 18 | 12 | CTX-M-15 / OXA-1 | ESBL + OXA-1 |
| 97 | 8 | 17 | 20 | 8 | CTX-M-15 / OXA-1 | ESBL + OXA-1 |
| 99 | 16 | 13 | 17 | 24 | OXA-1 / TEM-1 | OXA |
| 100 | 1 | 25 | 28 | 1.5 | None | None |
| 101 | 1 | 25 | 28 | 0.75 | TEM-1 | None |
| 102 | 1 | 23 | 26 | 2 | TEM-1 | None |
| 103 | 8 | 15 | 19 | 24 | TEM-40(IRT) | None |
| 105 | 8 | 18 | 22 | 6 | TEM-1 | None |
| 108 | 1 | 24 | 27 | 1 | TEM-1 | None |
| 109 | 1 | 25 | 28 | 1.5 | TEM-1 | None |
| 110 | 1 | 24 | 27 | 1.5 | TEM-1 | None |
| 111 | 4 | 22 | 25 | 4 | TEM-1 | None |
| 113 | 1 | 25 | 29 | 1.5 | None | None |
| 117 | 1 | 21 | 24 | 2 | CTX-M-1 / TEM-1 | ESBL |
| 118 | 1 | 25 | 28 | 1.5 | TEM-1 | None |
| 119 | 2 | 22 | 25 | 2 | TEM-1 | None |
| 122 | 2 | 23 | 26 | 4 | None | None |
| 123 | 1 | 25 | 28 | 2 | None | None |
| 124 | 8 | 15 | 19 | 16 | OXA-1 | OXA |
| 125 | 8 | 17 | 20 | 8 | OXA-1 / TEM-1 | OXA |
| 126 | 8 | 17 | 20 | 8 | OXA-1 / TEM-1 | OXA |
| 134 | 8 | 20 | 24 | 6 | None | None |
| 156 | 2 | 21 | 24 | 2 | CTX-M-9 / TEM-1 | ESBL |
| 164 | 32 | 17 | 21 | 12 | OXA-1 / TEM-1 | OXA |
| 169 | 8 | 16 | 20 | 8 | OXA-1 | OXA |
| 172 | 2 | 18 | 22 | 8 | OXA-1 | OXA |
| 206 | 4 | 15 | 19 | 12 | OXA-1 | OXA |
| 211 | 32 | 16 | 19 | 12 | OXA-1 | OXA |
| 212 | 4 | 18 | 22 | 12 | TEM-40(IRT) | None |
| 222 | 1 | 25 | 28 | 1.5 | None | None |
| 223 | 2 | 23 | 27 | 3 | None | None |
| 236 | 4 | 18 | 22 | 4 | CTX-M-1 / OXA-1 | ESBL + OXA-1 |
| 239 | 1 | 27 | 29 | 1.5 | None | None |
| 244 | 1 | 27 | 30 | 1 | None | None |
| 247 | 16 | 14 | 19 | 16 | CTX-M-1 / OXA-1 | ESBL + OXA-1 |
| 249 | 8 | 17 | 20 | 8 | OXA-1 | OXA |
| 286 | 16 | 17 | 20 | 8 | OXA-1 / TEM-1 | OXA |
| 301 | 16 | 17 | 21 | 12 | OXA-1 / TEM-1 | OXA |
| 308 | 8 | 17 | 20 | 12 | CTX-M-1 / OXA-1 | ESBL + OXA-1 |
| 309 | 8 | 16 | 20 | 8 | CTX-M-15 / OXA-1 | ESBL + OXA-1 |
| 328 | 2 | 20 | 26 | 4 | None | None |
| 336 | 8 | 17 | 21 | 8 | CTX-M-15 / OXA-1 | ESBL + OXA-1 |
| 346 | 16 | 16 | 20 | 16 | OXA-1 | OXA |
| 348 | 1 | 25 | 28 | 1.5 | None | None |
| 349 | 64 | 17 | 15 | 32 | OXA-1 | OXA |
| 364 | 2 | 22 | 25 | 3 | None | None |
| 365 | 4 | 23 | 26 | 1.5 | TEM-1 | None |
| 400 | 16 | 15 | 19 | 16 | OXA-1 / TEM-1 | OXA |
| 401 | 8 | 16 | 20 | 16 | CTX-M-1 / OXA-1 | ESBL + OXA-1 |
| 412 | 8 | 17 | 22 | 8 | CTX-M-15 / OXA-1 | ESBL + OXA-1 |

BMD: broth microdilution; DD: disc diffusion, none: no ESBL/OXA-1

**Figure S1. Quality control.**


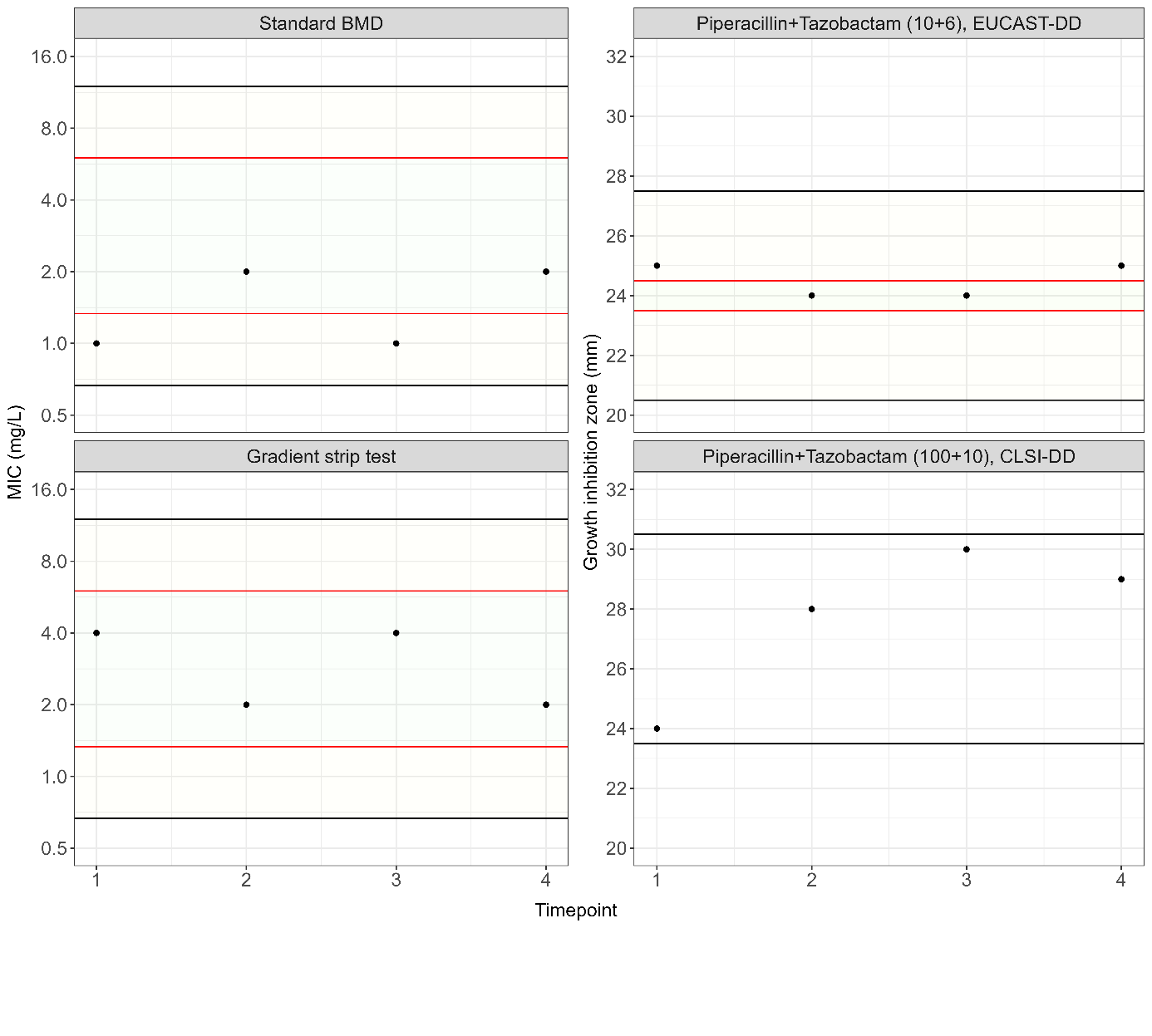
MIC values obtained by broth microdilution (BMD) and gradient strip test, and inhibition zone diameters from disc diffusion (DD) using EUCAST (30 + 6 µg) and CLSI (100 + 10 µg) piperacillin-tazobactam discs for *E. coli* ATCC 25922. Replica numbers are displayed on the x-axis; MIC values and inhibition zone diameters on the y-axis. For standard BMD, gradient strip test and ed and black horizontal lines indicate the EUCAST quality control target value and acceptable range, respectively. black horizontal lines indicate the EUCAST quality control target value and acceptable range.

**References**

1. Mancini S, Marchesi M, Imkamp F *et al.* Population-based inference of aminoglycoside resistance mechanisms in *Escherichia coli*. *EBioMedicine* 2019; **46**: 184-92.
